# The influence of a long-term growth hormone treatment on lipid and glucose metabolism: a randomized trial in short Japanese children born small for gestational age

**DOI:** 10.1186/s13633-016-0036-4

**Published:** 2016-10-26

**Authors:** Reiko Horikawa, Toshiaki Tanaka, Hiromi Nishinaga, Yoshihisa Ogawa, Susumu Yokoya

**Affiliations:** 1 Division of Endocrinology and Metabolism, National Center for Child Health and Development, 2-10-1 Okura, Setagaya-ku, Tokyo, 157-8535 Japan; 2 Tanaka Growth Clinic, 2-36-7 Yoga, Setagaya-ku, Tokyo, 158-0097 Japan; 3 CMR Development Division, Novo Nordisk Pharma Ltd., 2-1-1 Marunouchi, Chiyoda-ku, Tokyo, 100-0005 Japan; 4 Department of Medical Subspecialties, National Center for Child Health and Development, 2-10-1 Okura, Setagaya-ku, Tokyo, 157-8535 Japan

**Keywords:** Growth hormone, GH therapy, Small for gestational age, Short stature, Norditropin®, Japanese, Lipid metabolism, Glucose metabolism, Insulin resistance

## Abstract

**Background:**

Long-term growth hormone (GH) treatments in short children born small for gestational age (SGA) restore lipid metabolism, but also increase insulin resistance. The aim of this study was to evaluate the influence of long-term GH therapy on lipid and glucose metabolism as well as its dose dependency in short Japanese children born SGA.

**Methods:**

Eighty Japanese children with a short stature who were born SGA participated in this study; 65 were treated with fixed GH doses of 0.033 (low) or 0.067 (high) mg/kg/day for 260 weeks; 15 were untreated controls in the first year and were randomized to one of the two treatment groups at week 52. Serum cholesterol, glucose and insulin levels were regularly measured. An oral glucose tolerance test (OGTT) was conducted annually.

**Results:**

The mean age at the start of GH therapy was approximately 5.3 years. Serum total cholesterol (TC) and low-density lipoprotein cholesterol (LDL-C) in the high dose group significantly decreased over time during GH therapy. In both dose groups for TC, and in the high dose group for LDL-C, the higher the baseline values, the greater the decrease after 260 weeks. The rate of the decrease observed after 260 weeks in patients with high LDL-C levels was greater in the high dose group. Based on the results of OGTT, no patient was classified as being diabetic; however, annual increases were observed in post-OGTT insulin levels. After 260 weeks, the homeostasis model assessment as an index of insulin resistance (HOMA-IR) increased, suggesting that insulin resistance developed over time with the GH treatment, while 36.6 % of the subjects entered puberty.

**Conclusions:**

Long-term continuous GH treatment for children born SGA may have a potentially beneficial effect on several parameters in lipid metabolism and does not adversely affect glucose metabolism.

**Trial registration:**

GHLIQUID-1516, GHLIQUID-1517, Japan Pharmaceutical Information Center Clinical trial registration: JapicCTI-050132. Registered 13 September 2005. Retrospectively registered. JapicCTI-050137. Registered 13 September 2005. Retrospectively registered. ClinicalTrials.gov trial registration: NCT00184717. Registered 13 September 2005. Retrospectively registered.

## Background

Small for gestational age (SGA) is defined as birth weight and length below the 10th percentile for gestational age in Japan [1]. Approximately 10 % of children born SGA will not achieve catch-up growth by 2 years of age, and will remain short throughout their lives if left untreated [2–4].

A previous study reported that low birth weight was associated with the development of type 2 diabetes mellitus, hypertension, and hyperlipidemia in adulthood [5]. Furthermore, rapid catch-up in the weight of these children has been suggested to increase the risk of various medical conditions [6, 7].

The mechanisms underlying these risks have been examined, with increases in insulin resistance and/or intra-abdominal fat being implicated in this phenomenon [8]. Increases in insulin resistance may cause impairments in the growth hormone (GH)-insulin-like growth factor (IGF)-binding protein axis or be a consequence of these impairments [9].

GH treatments have been approved for the treatment of children with short stature born SGA without spontaneous catch-up growth. GH therapy has been suggested to provide additional, long-term metabolic benefits, thereby mitigating the metabolic consequences of being born SGA, while still facilitating early age-appropriate catch-up growth [10]. On the other hand, GH treatments have been reported to increase insulin resistance with the over-secretion of insulin occurring to compensate.

This was a 260 week (5 year) clinical study investigating the effect of long-term GH therapy in Japanese patients with SGA-related short stature. Height efficacy and safety data [11] and the beneficial effects on metabolic parameters (glucose, insulin, total cholesterol [TC], low-density lipoprotein cholesterol [LDL-C], high-density lipoprotein cholesterol [HDL-C]) [12] in these patients have been reported previously. In the present analysis, we examined the relationship between baseline status and changes of lipid metabolism-associated parameters after long-term (260 weeks) GH therapy, in addition to effects on other metabolic parameters including glycated hemoglobin A_1c_ (HbA_1c_), blood glucose and insulin levels before and after an oral glucose tolerance test (OGTT), homeostasis model assessment as an index of insulin resistance (HOMA-IR), and insulinogenic index.

## Methods

### Patients

The study population comprised 80 short children born SGA, aged 3–8 years, who were randomly assigned to two groups receiving a low or high dose (0.033 or 0.067 mg/kg/day) of GH (*n* = 31 and 34, respectively). A control group (no treatment, *n* = 15) was established during the first 52 weeks (1 year) and compared with the GH therapy groups. Patients from the control group were randomized to one of the treatment groups at week 52. Further details concerning inclusion and exclusion criteria have been described previously [11].

### Study preparations

Packaging of trial products were indistinguishable from one another and the doctors and the patients were blinded to their group allocation. Both GH doses were injected using a GH injection device (NordiPen® 5 and PenNeedle®; Novo Nordisk A/S) to maintain blinding.

### Study design

This study involved a 156-week extension of a 104-week (260 weeks in total) multicenter, randomized, double-blind, parallel-group trial investigating the efficacy and safety of two doses of GH. In patients assigned to receive long-term GH therapy, GH was subcutaneously injected daily before bedtime. In those assigned to the control group (52 weeks), follow-up alone was performed without GH. The trial was performed between July 2003 and December 2009. Patient visits were planned at 13-week intervals with a ±14-day window, and 15 visits were scheduled over the course of the 260-week trial.

Metabolic parameters assessed included: TC, LDL-C, HDL-C, HbA_1c_, blood glucose and insulin levels before and after an OGTT, HOMA-IR, and insulinogenic index (Δinsulin 0–30 min/Δglucose 0–30 min).

On the basis of the results of OGTT (1.75 g/kg, maximum 75 g), individual patients were classified into three types: normal, glucose intolerant, and diabetic, according to the criteria for hyperglycemia established by the Japan Diabetes Society [13, 14]. Patients were categorized as glucose intolerant if they had a fasting blood glucose level of 110–125 mg/dL and/or a value of 140–199 mg/dL at 120 min after OGTT, or a blood glucose level of 180 mg/dL or more 60 min after OGTT. Changes in body weight and body mass index (BMI) SDS were also reported.

### Statistical analysis

Summary statistics (mean with SD) were calculated for baseline patient demographics. A closed testing procedure was applied for analyses at 52 weeks since two hypotheses (low or high dose group vs. no treatment group) were tested at 52 weeks. Changes from baseline for the parameters of lipid metabolism were analyzed using a 2-sided *t*-test based on t-distribution for each treatment group. A significance level of 5 % was used, and confidence intervals (CI) were constructed with a confidence coefficient of 95 %. Simple correlation analysis was used to evaluate the relationship between baseline and change in the parameters of lipid metabolism, between HOMA-IR and BMI SDS at 260 weeks, and between HbA_1c_ and BMI SDS at 260 weeks.

## Results

### Patient demographics

Baseline patient demographics are outlined in Tables 1 and 2. No significant differences were observed in patient backgrounds at baseline among the three groups; however, the proportion of boys was higher in the low and high dose groups than in the no treatment group. At treatment start, the mean age, height SDS, and IGF-I SDS were approximately 5.3 years, –3.0, and –0.7, respectively.

**Table 1:** Patient demographics [Mean ± SD]

	No treatment		0.033 mg/kg/day		0.067 mg/kg/day	
		n		n		n
Gender		15		31		34
Male, %	46.7	7	64.5	20	58.8	20
Female, %	53.3	8	35.5	11	41.2	14
Chronological age, years	5.32 ± 1.38	15	5.34 ± 1.46	31	5.27 ± 1.15	34
Height, cm	94.6 ± 8.6	15	95.9 ± 8.4	31	94.9 ± 7.3	34
Height SDS	-2.92 ± 0.53	15	-2.95 ± 0.64	31	-2.90 ± 0.67	34
BMI	14.07 ± 1.40	15	14.42 ± 1.26	31	14.16 ± 1.24	34
IGF-I, ng/mL	115.39 ± 51.01	15	117.25 ± 49.13	31	118.20 ± 49.10	34
IGF-I SDS	-1.03 ± 1.50	15	-0.75 ± 1.06	31	-0.63 ± 1.21	34
Insulin, μU/mL	2.6 ± 1.7	10	3.6 ± 2.1	26	3.2 ± 1.9	28
Glucose, mg/dL	79.8 ± 6.3	15	82.4 ± 10.4	31	78.2 ± 11.5	34
HbA_1c_ (NGSP), %	5.08 ± 0.32	15	5.10 ± 0.29	31	5.00 ± 0.19	34
TC, mg/dL	173.5 ± 24.2	15	166.9 ± 29.8	31	181.4 ± 24.2	34
LDL-C, mg/dL	96.7 ± 18.9	15	94.8 ± 26.2	31	104.7 ± 25.0	34
HDL-C, mg/dL	60.4 ± 15.9	15	56.9 ± 9.6	31	61.9 ± 12.0	34

*SD* standard deviation, *SDS* standard deviation score, *BMI* body mass index, *IGF-I* insulin-like growth factor-1, *HbA*_*1c*_ glycated hemoglobin A1c, *NGSP* National Glycohemoglobin Standardization Program, *TC* total cholesterol, *LDL-C* low-density lipoprotein cholesterol, *HDL-C* high-density lipoprotein cholesterol

**Table 2:** Patient numbers evaluated as being the glucose intolerant type of diabetes mellitus [n (%)]

	0.033 mg/kg/day					0.067 mg/kg/day				
	Total	Glucose intolerant type	Time point evaluated as the glucose intolerant type			Total	Glucose intolerant type	Time point evaluated as the glucose intolerant type		
			Before	60 min^a^	120 min			Before	60 min*	120 min
Baseline	31 (100 %)	3 (9.7 %)	1 (3.2 %)	0 (0.0 %)	2 (6.5 %)	34 (100 %)	1 (2.9 %)	0 (0.0 %)	0 (0.0 %)	1 (2.9 %)
52 weeks	29 (100 %)	3 (10.3 %)	0 (0.0 %)	2 (6.9 %)	1 (3.4 %)	34 (100 %)	3 (8.8 %)	0 (0.0 %)	3 (8.8 %)	0 (0.0 %)
104 weeks	29 (100 %)	2 (6.9 %)	0 (0.0 %)	0 (0.0 %)	2 (6.9 %)	32 (100 %)	2 (6.3 %)	0 (0.0 %)	0 (0.0 %)	2 (6.3 %)
156 weeks	25 (100 %)	1 (4.0 %)	0 (0.0 %)	0 (0.0 %)	1 (4.0 %)	29 (100 %)	2 (6.9 %)	0 (0.0 %)	0 (0.0 %)	2 (6.9 %)
208 weeks	25 (100 %)	0 (0.0 %)	0 (0.0 %)	0 (0.0 %)	0 (0.0 %)	28 (100 %)	3 (10.7 %)	0 (0.0 %)	2 (7.1 %)	1 (3.6 %)
260 weeks	23 (100 %)	1 (4.3 %)	1 (4.3 %)	0 (0.0 %)	0 (0.0 %)	27 (100 %)	4 (14.8 %)	0 (0.0 %)	1 (3.7 %)	3 (11.1 %)

^a^Patients with a blood glucose level of 180 mg/dL or more 60 min after OGTT were regarded as being glucose intolerant [14]. OGTT, oral glucose tolerance test

### Influence on lipid metabolism

**Fig. 1:**
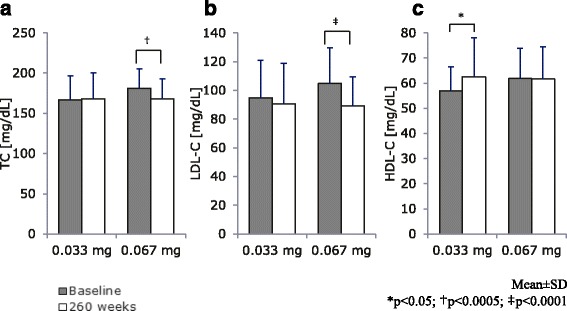
(**a**) TC, (**b**) LDL-C, and (**c**) HDL-C values at baseline and week 260. TC, total cholesterol; LDL-C, low-density lipoprotein cholesterol; HDL-C, high-density lipoprotein cholesterol

We analyzed lipid parameters at the start of GH therapy and after 260 weeks. In the low dose group, no significant differences were noted in the mean TC and LDL-C levels between the two time points (Fig. 1a and b, respectively). In the high dose group, a significant decrease was observed after 260 weeks (*p* < 0.0005 and *p* < 0.0001, respectively). In the low dose group, the mean HDL-C level after 260 weeks was significantly higher than at the start of GH therapy (*p* < 0.05, Fig. 1c), whereas no significant difference was found in the high dose group.

**Fig. 2:**
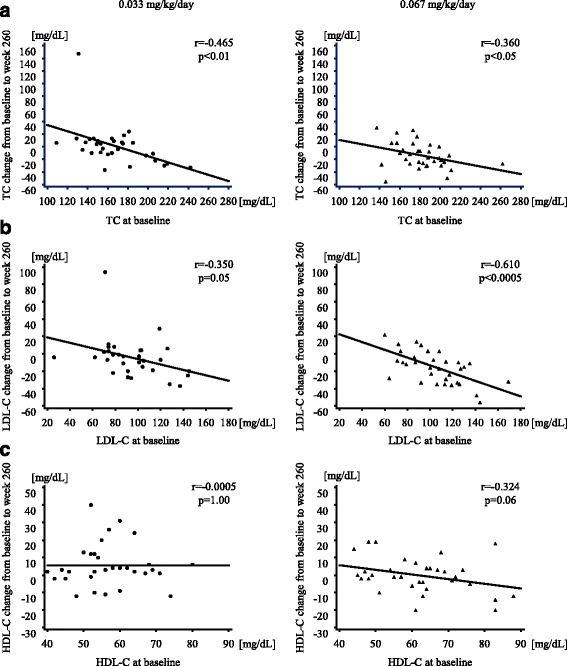
Scatter plots of (**a**) TC, (**b**) LDL-C, and (**c**) HDL-C values at baseline, and changes from baseline to week 260. TC, total cholesterol; LDL-C, low-density lipoprotein cholesterol; HDL-C, high-density lipoprotein cholesterol

Correlations between the baseline values and changes from baseline to 260 weeks in TC, LDL-C, and HDL-C levels are shown in Fig. 2. Changes in TC levels were negatively correlated with baseline values in the two dose groups (Fig. 2a). A similar correlation was observed between LDL-C levels at the start of GH therapy and the change noted after 260 weeks (Fig. 2b). This correlation was particularly strong in the high dose group. No correlation was detected between HDL-C levels at the start of GH therapy and the change noted after 260 weeks in either group (Fig. 2c).

### Influence on glucose metabolism

**Fig. 3:**
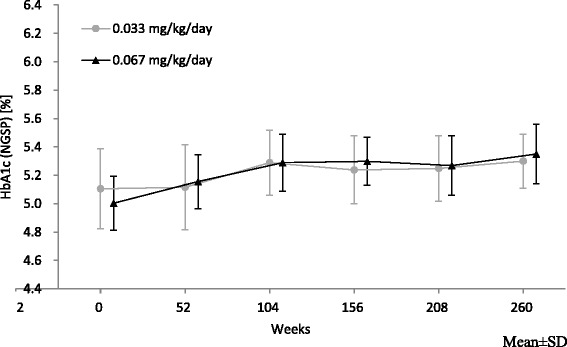
HbA_1c_ (NGSP) during the study period. HbA_1c_, glycated hemoglobin A_1c_; NGSP, National Glycohemoglobin Standardization Program

In the two dose groups, HbA_1c_ (National Glycohemoglobin Standardization Program [NGSP]) values increased until 104 weeks after the start of GH therapy, and remained constant thereafter (Fig. 3). In the low and high dose groups, the mean HbA_1c_ values at 260 weeks after the start of GH therapy were 5.29 and 5.33 %, respectively, which were within the normal range (4.6–6.2 %). Furthermore, no patient showed an HbA_1c_ value beyond the standard value range during the study period (260 weeks).

**Fig. 4:**
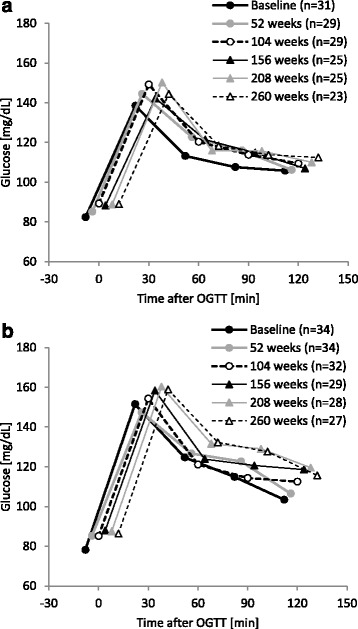
Changes in glucose after OGTT. **a** 0.033 mg/kg/day; **b** 0.067 mg/kg/day. OGTT, oral glucose tolerance test

**Fig. 5:**
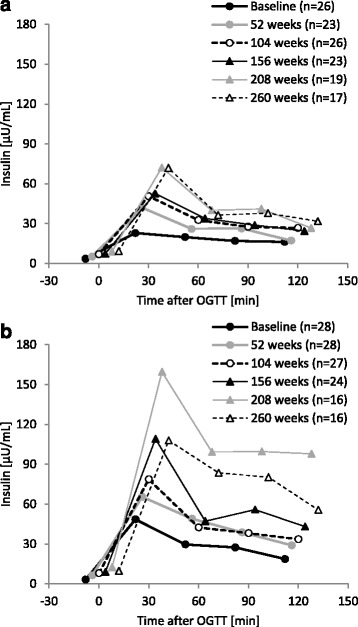
Changes in insulin after OGTT. **a** 0.033 mg/kg/day; **b** 0.067 mg/kg/day. OGTT, oral glucose tolerance test

No marked changes were noted in the blood glucose response to OGTT during 260 weeks of the treatment in either group (Fig. 4). Regarding the influence of long-term GH therapy on insulin levels after OGTT, the peak value in the high dose group was slightly higher than that in the low dose group (Fig. 5).

Although no marked changes were observed in fasting blood glucose levels, HbA_1c_ values slightly increased from 5.10 to 5.29 % and from 5.00 to 5.33 % in the low and high dose groups, respectively. Two patients were classified as being glucose intolerant based on fasting blood glucose levels. GH therapy had not been administered to one of these patients. In the low and high dose groups, 95.7 and 85.2 % of patients were classified as being normal 260 weeks after the start of GH therapy, respectively. No patients were classified as being diabetic at any observation point.

An increase was noted in the mean HOMA-IR 260 weeks after the start of GH therapy (Table 3). However, no significant difference was noted in this increase that was related to the dose administered. Twenty-two patients (36.6 %) entered puberty during the study period (7 [35 %] in the low dose group and 15 [37.5 %] in the high dose group).

**Table 3:** HOMA-IR [Mean ± SD]

	0.033 mg/kg/day n = 20	0.067 mg/kg/day n = 16
Baseline	0.720 ± 0.465	0.646 ± 0.498
260 weeks	2.154 ± 1.682	1.895 ± 1.197

*SD* standard deviation, *HOMA-IR* homeostatic model assessment as an index of insulin resistance

No correlations were found between the HOMA-IR and BMI SDS or between the HbA_1c_ and BMI SDS after 260 weeks of GH therapy (data not shown).

After 260 weeks of GH therapy, the insulinogenic index increased from 0.45 to 1.31 and 0.52 to 1.24 in the low and high dose groups, respectively; however, no dose-related differences were observed.

### Withdrawals

Two patients discontinued treatment and withdrew from the study. One patient from the 0.033 mg/kg/day group withdrew after 151 weeks due to IgA nephropathy. The event was classed as a serious adverse event, moderate in severity and assessed as possibly related to trial product. One patient from the no treatment group/0.033 mg/kg/day withdrew after 212 weeks due to hyperinsulinemia. The event was mild in severity and assessed as possibly related to trial product.

## Discussion

### Influence on lipid metabolism

In the high dose group, significant decreases were found in TC and LDL-C levels 260 weeks after the start of GH therapy (Fig. 1). Mean TC decreased, especially in the high dose group, and this change was attributed to a decrease in LDL-C. The baseline status may have affected these decreases, because baseline mean values were significantly higher in male patients in the high dose group, leading to more significant decreases with the treatment, suggesting there might be a confounding factor (data not shown). TC and LDL-C levels were slightly lower in female patients. Since TC levels normally increase with puberty [15], the degree of decrease in TC observed may have been attenuated by pubertal hormonal changes. In the low dose group, a significant increase was observed in HDL-C levels, whereas no significant decreases were noted in TC and LDL-C levels. However, the clinical significance of this result currently remains unclear and awaits further investigation.

After 260 weeks, decreases in TC in the two dose groups and LDL-C in the high dose group correlated with baseline values, indicating that the greater the baseline value, the greater the decrease observed after the treatment (Fig. 2). In both groups, a higher TC was more likely to decrease during GH therapy. This result is consistent with previous findings [16, 17], suggesting that long-term GH treatment has a favorable lowering effect on cholesterol, especially in those with higher cholesterol levels due to genetic and/or environmental backgrounds. No correlation was observed between HDL-C levels at the start of the study and changes after 260 weeks of GH therapy. One patient had markedly higher TC and LDL-C levels than those of the other patients. This patient had a history of IgA nephropathy, which may have elevated TC and LDL-C levels [18].

### Influence on glucose metabolism

Post-OGTT insulin levels increased with each year of long-term GH treatment, particularly in the high dose group (Fig. 5). It is possible that the increase in HOMA-IR levels in the final year of GH therapy was a result of patients transitioning into puberty. Overall, 35 % of patients in the low dose group and 37.5 % of patients in the high dose group entered puberty during the study period. The increase in post-OGTT insulin levels is consistent with previous findings in which patients became relatively resistant to insulin after the start of GH therapy [19]. Fasting insulin levels have been shown to increase with age [15]; therefore, the results obtained in this study reflected physiological changes. Previous studies demonstrated that bioactive IGF-I levels increased through decreases in IGF-binding protein in the presence of hyperinsulinemia [20]. Furthermore, an increase in insulin levels has been suggested to promote growth [21].

After long-term GH therapy (260 weeks), the HOMA-IR (Table 3) and insulinogenic index increased. However, no significant differences were observed between the two dose groups, indicating no dose dependency. These results also demonstrated that the GH dose of 0.033 mg/kg/day was already greater relative to GH secreted from the pituitary in normal physiology. Patients with a HOMA-IR of 2.5 or higher are regarded as being resistant to insulin [13, 14], and 25 % of patients in this study (low dose group: 5 out of 20 patients, high dose group: 4 out of 16 patients) exhibited insulin resistance after 260 weeks of GH therapy. These results suggest that, although the dose may be increased in accordance with the responsiveness of individual patients to GH therapy, dose elevations within these limits do not influence glucose tolerance.

No correlations were observed between the HOMA-IR and BMI SDS or between the HbA_1c_ and BMI SDS after 260 weeks of GH therapy. The mean BMI SDS in this study was below 0 SD during the study period and our patients did not include those with a very high/low BMI. A larger number of patients need to be analyzed in future studies in order to more clearly elucidate the relationship between BMI and glucose metabolism in GH-treated SGA.

## Conclusions

This study investigated the effect of long term continuous GH treatment in children born SGA on lipid and glucose metabolism. In the high dose group, TC and LDL-C levels decreased significantly over time during the GH therapy. Baseline values correlated with the reductions after 260 weeks in both groups for TC and in the high dose group for LDL-C. This result suggests that the long-term GH treatment used had favorable lowering effects on cholesterol, especially in those with higher cholesterol levels. HbA_1c_ values slightly increased, and post-OGTT insulin levels increased with each year of GH treatment; however, no marked changes were noted in the blood glucose response to OGTT during the 260 weeks of treatment. In healthy adolescents, decreased insulin sensitivity observed during puberty is compensated for by an increase in insulin secretion, hence the increase in HOMA-IR levels observed in the final year of GH therapy may have been a result of patients transitioning into puberty.

The results of this study suggest long-term continuous GH treatment for children born SGA may have potentially beneficial effects on several parameters of lipid metabolism and does not have an adverse effect on glucose metabolism.

## Appendix

### Institutional review boards providing approval for the present study

Pharmaceuticals Clinical Study Institutional Review Board, Asahikawa Medical College Hospital

Institutional Review Board, Kushiro Red Cross Hospital

Pharmaceuticals Clinical Study Institutional Review Board, Hirosaki University School of Medicine Hospital

Institutional Review Board, Igarashi Children’s Clinic

Institutional Review Board, Niigata City General Hospital

Pharmaceuticals・Medical Devices Clinical Study Institutional Review Board, Niigata University Medical and Dental Hospital

Clinical Study Institutional Review Board, Gunma University Hospital

Institutional Review Board, Dokkyo University School of Medicine Hospital

Pharmaceuticals Entrusted Clinical study Institutional Review Board

Institutional Review Board, Saitama Medical School Hospital

Institutional Review Board, National Center for Child Health and Development

Institutional Review Board, Toranomon Hospital

Institutional Review Board, Keio University Hospital

Institutional Review Board, Tokyo Metropolitan Ohtsuka Hospital

Institutional Review Board, Tokyo Metropolitan Kiyose Children’s Hospital

Pharmaceuticals Clinical Study Institutional Review Board, University of Yamanashi Hospital

Institutional Review Board, National Hospital Organization Nagano Hospital

Institutional Review Board, Juntendo Shizuoka Hospital

Institutional Review Board, Hamamatsu Medical School Hospital

Institutional Review Board, Fujita Health University Hospital

Institutional Review Board, Toyohashi Municipal Hospital

Pharmaceuticals Clinical Study Institutional Review Board, Nagoya City University Hospital

Institutional Review Board, Japanese Red Cross Nagoya First Hospital

Institutional Review Board, West Medical Center Jouhoku Municipal Hospital, City of Nagoya

Pharmaceuticals Entrusted Clinical Study Institutional Review Board, Osaka University Hospital

Institutional Review Board, Osaka Medical Center and Research Institute for Maternal and Child Health

Entrusted Clinical Study Institutional Review Board, Osaka City General Hospital

Institutional Review Board, Kansai Medical University Kori Hospital

Institutional Review Board, Osaka Prefecture Medical Association

Pharmaceuticals Clinical Study Institutional Review Board, Kagawa University Hospital

Institutional Review Board, Okayama University Hospital

Institutional Review Board, Hiroshima Red Cross Hospital & Atomicbomb Survivors Hospital

Institutional Review Board, Hiroshima Prefectural Hospital

Institutional Review Board, Tottori University Hospital

Institutional Review Board, Kumamoto University Hospital

Institutional Review Board, Kumamoto University Hospital

Institutional Review Board, Miyazaki University Hospital

Institutional Review Board, Ichinomiya Municipal Hospital

Pharmaceuticals Clinical Study Institutional Review Board, Fukui University Hospital

Institutional Review Board, Kyushu Kosei Nenkin Hospital

Clinical Study Institutional Review Board, Fukuoka University Hospital

Institutional Review Board, Tohoku University Hospital

Institutional Review Board, Tendo Municipal Hospital

Institutional Review Board, Osaka Kosei Nenkin Hospital

## Abbreviations

BMI: Body mass index
GH: Growth hormone
HbA_1c_: Glycated hemoglobin A_1c_
HDL-C: High-density lipoprotein cholesterol
HOMA-IR: Homeostatic model assessment as an index of insulin resistance
IGF-I: Insulin-like growth factor-1
LDL-C: Low-density lipoprotein cholesterol
NGSP: National Glycohemoglobin Standardization Program
OGTT: Oral glucose tolerance test
SDS: Standard deviation score
SGA: Small for gestational age
TC: Total cholesterol

## References

[1] Tanaka T, Yokoya S, Nishi Y, Hasegawa Y, Yorifuji T, Fujieda K (2007). Management of short children born small for gestational age. J Jpn Pediatr Soc.

[2] Karlberg J, Albertsson-Wikland K (1995). Growth in full-term small-for-gestational-age infants: from birth to final height. Pediatr Res.

[3] Hokken-Koelega AC, De Ridder MA, Lemmen RJ, Den Hartog H, De Muinck Keizer-Schrama SM, Drop SL (1995). Children born small for gestational age: do they catch up?. Pediatr Res.

[4] Itabashi K, Mishna J, Tada H, Sakurai M, Nanri Y, Hirohata Y (2007). Longitudinal follow-up of height up to five years of age in infants born preterm small for gestational age; comparison to full-term small for gestational age infants. Early Hum Dev.

[5] Barker DJ, Hales CN, Fall CH, Osmond C, Phipps K, Clark PM (1993). Type 2 (non-insulin-dependent) diabetes mellitus, hypertension and hyperlipidaemia (syndrome X): relation to reduced fetal growth. Diabetologia.

[6] Ong KK, Petry CJ, Emmett PM, Sandhu MS, Kiess W, Hales CN (2004). Insulin sensitivity and secretion in normal children related to size at birth, postnatal growth, and plasma insulin-like growth factor-I levels. Diabetologia.

[7] Soto N, Bazaes RA, Peña V, Salazar T, Avila A, Iñiguez G (2003). Insulin sensitivity and secretion are related to catch-up growth in small-for-gestational-age infants at age 1 year: results from a prospective cohort. J Clin Endocrinol Metab.

[8] Mericq V, Ong KK, Bazaes R, Pena V, Avila A, Salazar T (2005). Longitudinal changes in insulin sensitivity and secretion from birth to age three years in small- and appropriate-for-gestational age children. Diabetologia.

[9] Lebl J, Lebenthal Y, Kolouskova S, Steensberg A, Jons K, Kappelgaard AM (2011). Metabolic impact of growth hormone treatment in short children born small for gestational age. Horm Res Paediatr.

[10] van Dijk M, Bannink EM, van Pareren YK, Mulder PG, Hokken-Koelega AC (2007). Risk factors for diabetes mellitus type 2 and metabolic syndrome are comparable for previously growth hormone-treated young adults born small for gestational age (sga) and untreated short SGA controls. J Clin Endocrinol Metab.

[11] Tanaka T, Yokoya S, Seino Y, Togari H, Mishina J, Kappelgaard AM, Fujieda K (2011). Long-term efficacy and safety of two doses of growth hormone in short Japanese children born small for gestational age. Horm Res Paediatr.

[12] Kappelgaard AM, Kiyomi F, Horikawa R, Yokoya S, Tanaka T (2014). The impact of long-term growth hormone treatment on metabolic parameters in Japanese patients with short stature born small for gestational age. Horm Res Paediatr.

[13] Seino Y, Nanjo K, Tajima N, Kadowaki T, Kashiwagi A, Committee of the Japan Diabetes Society on the Diagnostic Criteria of Diabetes Mellitus (2010). Report of the committee on the classification and diagnostic criteria of diabetes mellitus. J Diabetes Investig.

[14] Japan Diabetes Society (2014). Treatment guide for diabetes 2014–2015.

[15] Matsui I, Nambu S, Baba S (1998). Evaluation of fasting serum insulin levels among Japanese school-age children. J Nutr Sci Vitaminol.

[16] Abs R, Feldt-Rasmussen U, Mattsson AF, Monson JP, Bengtsson BA, Góth MI (2006). Determinants of cardiovascular risk in 2589 hypopituitary GH-deficient adults – a KIMS database analysis. Eur J Endocrinol.

[17] Monson JP, Jönsson P, Koltowska-Häggström M, Kourides I (2007). Growth hormone (GH) replacement decreases serum total and LDL-cholesterol in hypopituitary patients on maintenance HMG CoA reductase inhibitor (statin) therapy. Clin Endocrinol (Oxf).

[18] Asami T, Hayakawa H, Ohkawa K, Uchiyama M (1999). Hypercholesterolemia and glomerular diseases in urinary screening of school children. Pediatr Nephrol.

[19] Sas T, Mulder P, Aanstoot HJ, Houdijk M, Jansen M, Reeser M, Hokken-Koelega A (2001). Carbohydrate metabolism during long-term growth hormone treatment in children with short stature born small for gestational age. Clin Endocrinol (Oxf).

[20] Yasunaga T, Furukawa S, Katsumata N, Horikawa R, Tanaka T, Tanae A, Hibi I (1998). Nutrition related hormonal changes in obese children. Endocr J.

[21] Messina JL (2011). Insulin as a growth-promoting hormone. Handbook of physiology. Supplement 24. The Endocrine System, Hormonal Control of Growth.

